# Seldom Differential of Dysuria in Northern America

**DOI:** 10.7759/cureus.6066

**Published:** 2019-11-03

**Authors:** Jad A Degheili, Jose M El-Asmar, Moustafa Moussally, Nassib Abou Heidar, Albert El Hajj

**Affiliations:** 1 Division of Urology, Department of Surgery, American University of Beirut Medical Center, Beirut, LBN

**Keywords:** schistosomiasis, bladder, genitourinary system, schistosoma, ova, urinary symptoms

## Abstract

Human schistosomiasis or bilharzia is a parasitic disease that highly impacts a country’s health and economic systems specifically when it affects individuals residing in underdeveloped countries. Daughter eggs produced by colonized Schistosoma can lead to multisystem immune-mediated response, one of them is an intravesicular granulomatous reaction leading to intramural lesions. Such outcome is directly related to the incubation of adult worms within the perivesical urogenital venous plexus. We hereby report an incidental discovery of calcified bladder wall lesions in a female patient residing in the United States for the last 27 years who presented with lower urinary tract symptoms. Despite a negative past medical history of schistosomiasis, intraoperative biopsies confirmed the presence of a calcified Schistosoma haematobium ova. Following that, a brief literature review of the pathogenesis and urogenital manifestations of Schistosoma is highlighted.

## Introduction

Schistosomiasis is a widespread infectious disease caused by a trematode parasite of the genus Schistosoma [1,2]. Unlike other trematodes, Schistosoma are dioecious fluke worms with distinct sexual dimorphism; they may reach 20 mm in length. Adult schistosomes are characterized by a cylindrical body with two terminal suckers that permit adequate suckling on the host’s blood and globulins via anaerobic glycolysis [1-3]. Furthermore, these organisms mate and deliver fertilized eggs within the mesenteric or perivesical venous plexuses of their human host, and these eggs will be either retained within the host’s system inducing infection or excreted via urine or feces [4].

Schistosomiasis, also referred to as bilharzia, is the second most common socioeconomically debilitating parasitic infection, after malaria. It affects approximately 240 million individuals around the world [1,5]. This disease is endemic to South America, Southeast Asia, parts of the Middle East, and Sub-Saharan Africa. Schistosoma haematobium is mainly present in Africa and the Middle East.

We hereby report a previously healthy middle-aged female who was incidentally diagnosed with genitourinary schistosomiasis upon investigation for mild lower urinary tract symptoms.

## Case presentation

A previously healthy 37-year-old female patient, currently residing in the United States, presented to our urology outpatient clinic complaining of dysuria of few weeks duration. She reports being born and raised in Ghana for the first 10 years of her life before migrating to the United States. During her time in Ghana, she admits to frequent swimming in fresh water lakes. Nevertheless, she denied any past medical history of schistosomiasis nor of lower urinary tract symptoms prior to this presentation 

Upon current presentation, a urine analysis was negative for any signs of infection or red blood cells and a complete blood count and creatinine tests were within normal range. Moreover, a kidney and pelvis ultrasound revealed diffuse thickening of the bladder wall, yet there was no presence of gross intramural lesions and the upper urinary tracts were normal.

**Figure 1:**
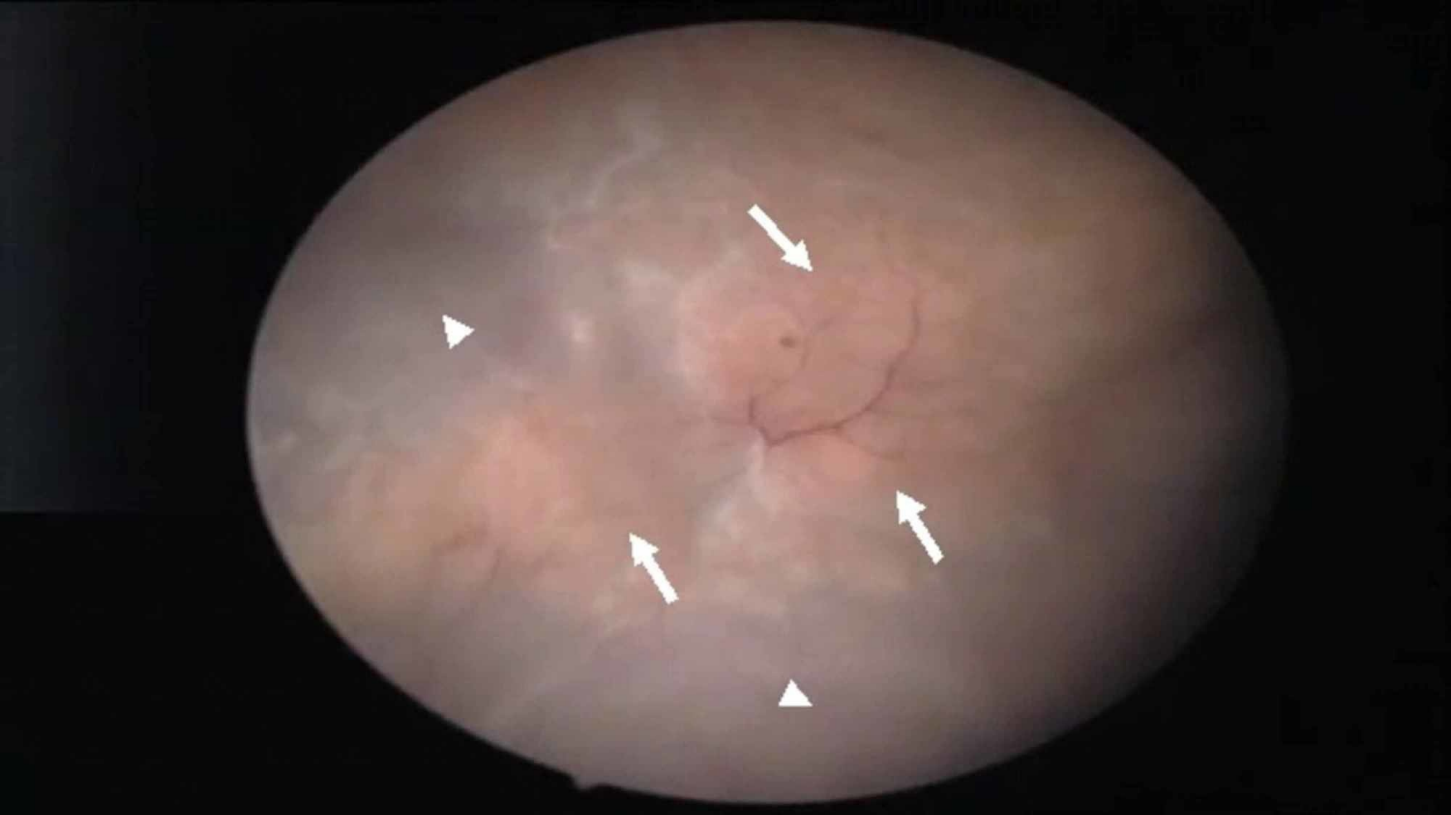
Cystoscopic view of the bladder revealing a yellowish and calcified sessile-like lesions (arrows) located at the dome and the right lateral wall of the bladder. No polypoid lesions were noted. Normal bladder wall urothelium is also seen (arrowheads).

Upon those findings, the decision was made to proceed with a cystoscopy and possible transurethral resection of bladder tumor. Polypoid lesions were not identified. However, a yellowish carpet of dense tissue was noted over the bladder dome and the right anterior bladder wall (Figure 1).

**Figure 2:**
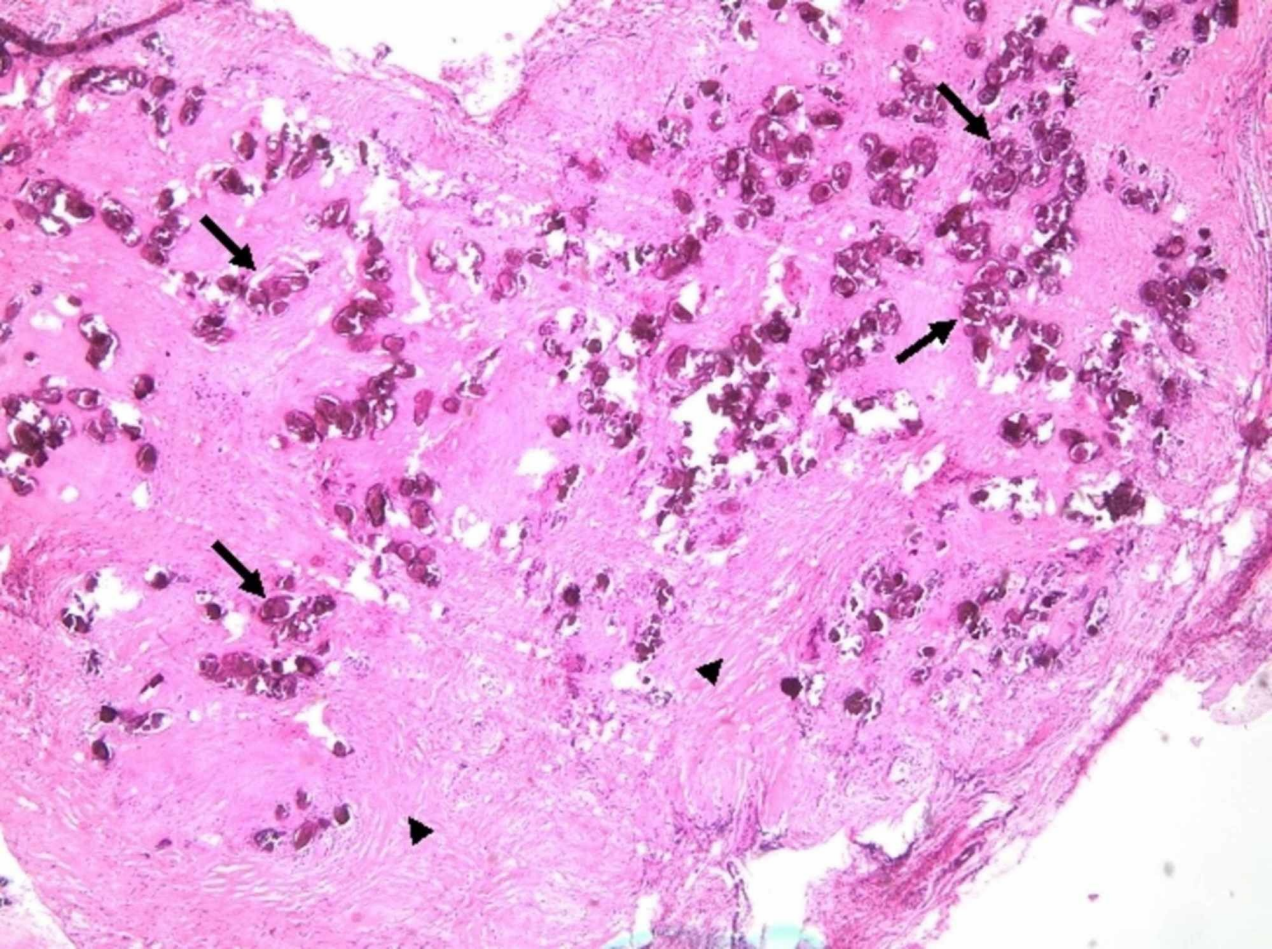
H&E staining of the bladder wall biopsies (x40) showing the presence of scattered helminth eggs of Schistosoma (arrows) within a milieu of smooth muscle cells (arrowheads) of the bladder and reactive urothelium. H&E, hematoxylin and eosin

**Figure 3:**
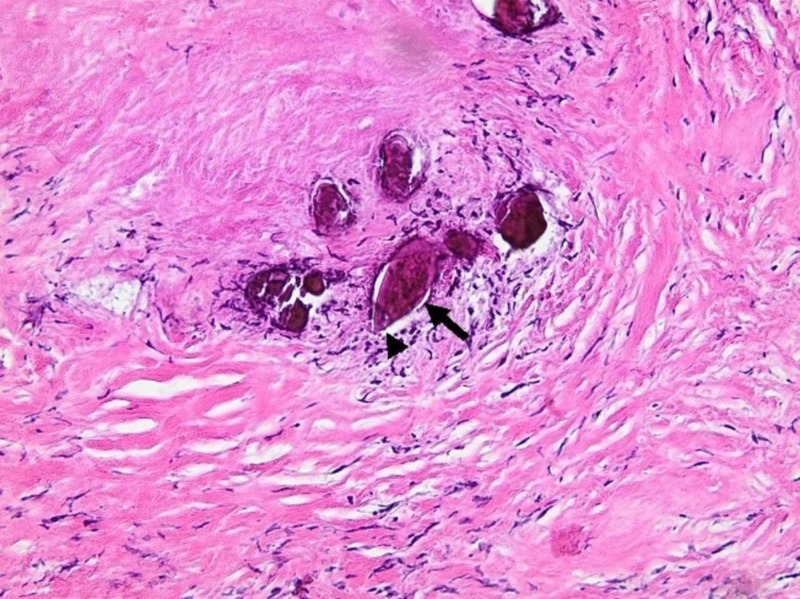
H&E staining of the bladder wall biopsies at higher magnification (x200) showing numerous calcified helminth eggs (arrow), with wide base and rounded tip, with the presence of a terminal spine (arrowhead). Each egg measured approximately 120 to 180 µm in length and 45 to 70 µm in width. H&E, hematoxylin and eosin

Biopsies were taken from both sites, and the pathology came out as a calcified parasite ova with a terminal spine, consistent with Schistosoma haematobium (Figures 2,3).

 

.

## Discussion

Among the various Schistosoma strains, Schistosoma haematobium is notorious for causing urogenital disease. After gaining entry into the human host through skin penetration, Schistosoma haematobium travels hematogenously to the liver where they mature into adult worms. Following that, the paired adult Schistosoma haematobium worms migrate to the pelvic venous plexus where they reside and clear their eggs into the host’s urinary bladder [1,3]. These eggs may result in a granulomatous immune response and chronic inflammation, which can lead to bladder wall ulcers, polyps, calcifications, or fibrosis [4,6,7]. As such, the presenting symptoms of urogenital schistosomiasis can be dysuric and/or hematuric in nature [3]. Moreover, parasitic eggs are excreted in urine and the microscopic visualization of eggs in urine can be used to diagnose active schistosomiasis [4]. However, egg excretion is not detectable in late chronic infections [1]. As such, many serologic tests have been recently used to diagnosis genitourinary schistosomiasis, yet their discussion is beyond the scope of this paper. Praziquantel is the drug of choice for the treatment of schistosomiasis with a cure rate ranging between 60% and 90% [4,8]. Praziquantel is effective for almost all species of schistosomiasis with some reported resistance according to their geographical location [8,9].

Gross hematuria may be present in up to 97% of untreated children exposed to genitourinary Schistosoma, while microscopic hematuria can be present in 41%-100% of cases [2]. The hematuria usually resolves after the age of 15 years, but complications pertaining to the upper urinary tracts such as hydroureter and hydronephrosis may still arise especially with the presence of granuloma formation near the ureteral orifices and bladder trigone.

Chronic urogenital schistosomiasis has been strongly associated with bladder cancer. The causality behind it arises from the chronic inflammatory state it creates leading to an excessive amount of oxidative stress, and hence bladder epithelium damage that induces tumorigeneses [10].

Radiologically, a bladder ultrasound reveals homogenous and wave-like bladder wall thickening in the acute phase [6,7]. In addition, hypervascular polyp-like lesions may be noted. Chronic phase of bladder schistosomiasis is characterized by the presence of linear calcifications that appear as shadow-casting echogenic foci in the bladder wall. To our knowledge, this is the first case of bladder schistosomiasis that is diagnosed more than 20 years post migration from the country of exposure. 

## Conclusions

Schistosoma of the genitourinary tract has been reported in patients inhabiting endemic areas of Schistosoma. Lower urinary tract symptoms secondary to Schistosoma bladder lesions may be evident. Praziquantel is still considered the most effective single dosage treatment. Symptom manifestations typically occur within weeks to months after exposure. Our patient’s cystoscopy revealed the presence of bladder lesions several years after trematodal exposure albeit no active infection.
